# Investigation of the hepatic development in the coculture of hiPSCs-derived LSECs and HLCs in a fluidic microenvironment

**DOI:** 10.1063/5.0041227

**Published:** 2021-05-10

**Authors:** Mathieu Danoy, Yannick Tauran, Stephane Poulain, Rachid Jellali, Johanna Bruce, Marjorie Leduc, Morgane Le Gall, Yuta Koui, Hiroshi Arakawa, Francoise Gilard, Bertrand Gakiere, Yukio Kato, Charles Plessy, Taketomo Kido, Atsushi Miyajima, Yasuyuki Sakai, Eric Leclerc

**Affiliations:** 1 CNRS UMI 2820, Laboratory for Integrated Micro Mechatronic Systems, Institute of Industrial Science, University of Tokyo, 4-6-1 Komaba, Meguro-ku, Tokyo 153-8505, Japan; 2 Department of Chemical System Engineering, graduate school of Engineering, University of Tokyo, 7-3-1, Hongo, Bunkyo-ku, Tokyo 113-8656, Japan; 3 Univ Lyon, Université Claude Bernard Lyon 1, Laboratoire des Multimatériaux et Interfaces, UMR CNRS 5615, F-69622 Villeurbanne, France; 4 RIKEN Center for Life Science Technologies, Division of Genomic Technologies, 1-7-22, Suehiro-cho, Tsurumi-ku, Yokohama, Kanagawa 230-0045, Japan; 5 Université de Technologie de Compiègne, CNRS, Biomechanics and Bioengineering, Centre de recherche Royallieu, CS 60319, 60203 Compiègne Cedex, Compiegne, France; 6 Plateforme protéomique 3P5, Université de Paris, Institut Cochin, INSERM, CNRS, F-75014 Paris, France; 7 Laboratory of Stem Cell Therapy, Institute for Quantitative Biosciences, The University of Tokyo, 1-1-1 Yayoi, Bunkyo-ku, Tokyo 113-0032, Japan; 8 Laboratory of Molecular Pharmacokinetics, Faculty of Pharmacy, Institute of Medical, Pharmaceutical and Health Sciences, Kanazawa University, Kakuma-machi, Kanazawa City, Ishikawa 920-1192, Japan; 9 Institute of Plant Sciences Paris-Saclay (IPS2), UMR 9213/UMR1403, CNRS, INRA, Université Paris-Sud, Université d'Evry, Université Paris-Diderot, Sorbonne Paris-Cité, Saclay Plant Sciences, Bâtiment 630, 91405 Orsay, France

## Abstract

Interactions between the different liver cell types are critical to the maintenance or induction of their function *in vitro*. In this work, human-induced Pluripotent Stem Cells (hiPSCs)-derived Liver Sinusoidal Endothelial Cells (LSECs) and Hepatocytes-Like Cells (HLCs) were cultured and matured in a microfluidic environment. Both cell populations were differentiated in Petri dishes, detached, and inoculated in microfluidic biochips. In cocultures of both cell types, the tissue has exhibited a higher production of albumin (3.19 vs 5.31 *μ*g/mL/10^6^ cells in monocultures and cocultures) as well as a higher inducibility CYP450 over monocultures of HLCs. Tubular-like structures composed of LSECs and positive for the endothelial marker PECAM1, as well as a tissue more largely expressing Stabilin-2 were detected in cocultures only. In contrast, monocultures exhibited no network and less specific endothelial markers. The transcriptomic analysis did not reveal a marked difference between the profiles of both culture conditions. Nevertheless, the analysis allowed us to highlight different upstream regulators in cocultures (SP1, EBF1, and GATA3) and monocultures (PML, MECP2, and NRF1). In cocultures, the multi-omics dataset after 14 days of maturation in biochips has shown the activation of signaling related to hepatic maturation, angiogenesis, and tissue repair. In this condition, inflammatory signaling was also found to be reduced when compared to monocultures as illustrated by the activation of NFKB and by the detection of several cytokines involved in tissue injury in the latter. Finally, the extracted biological processes were discussed regarding the future development of a new generation of human *in vitro* hepatic models.

## I. INTRODUCTION

The differentiation of hepatic cells and the maintenance of their phenotypes in *in vitro* cocultures of hepatocytes and non-parenchymal cells has been widely studied (Bale *et al.*, 2015). These efforts aim at reproducing the complex structure of the liver, comprised of a multitude of cell types in close interaction. In detail, the liver microvasculature is mostly composed of Liver Sinusoidal Endothelial Cells (LSECs) supported by Hepatic Stellate Cells (HSCs) in the space of Disse which separate them from hepatocytes (Arias, 2009). LSECs are known to exhibit fenestration which allows constant exchanges between hepatocytes and the blood. LSECs are also reported to be of importance in maintaining the quiescent state of HSCs, thus inhibiting the development of liver fibrosis (Poisson *et al.*, 2017). Alas, primary hepatocytes (Bale *et al.* 2015) and LSECs (Fraser *et al.*, 1995) are known to rapidly lose their phenotype and metabolic functions *in vitro* which consist an important remaining issue in *in vitro* modeling.

An alternative to the use of those cells lies in the development of human-induced Pluripotent Stem Cells (hiPSCs) for investigative and drug screening purposes. Several groups have described different strategies for the differentiation and maturation of hiPSCs into Hepatocytes-Like Cells (HLCs) (Spence and Wells, 2007; Si-Tayeb *et al.*, 2010; Kido *et al.*, 2015). Although those hiPSCs-derived cells presented advanced phenotypes in the form of albumin production and various CYP450 activity, their functionality remained quite far from mature hepatocytes. Similarly, hiPSCs have been differentiated toward LSECs phenotypes and have shown advanced liver-specific profiles (Koui *et al.*, 2017).

Once maturation has been achieved, those new cellular sources could be key to the development of complex *in vitro* models which could be used as a replacement for hepatocytes, one of the major bottlenecks in the pharmaceutical industry. Several strategies have been proposed to improve the *in vitro* maturation of tissue using tissue engineering approaches such as organoid reconstruction (Lee *et al.*, 2020), including hiPSCs-derived cells (Takebe *et al.*, 2017). The organ on chip technology was also used to build functional *in vitro* models (Zheng *et al.*, 2016; Liu *et al.*, 2019), which demonstrated the potential of the technology in liver applications (Jellali *et al.*, 2016; 2018) and hiPSCs-derived liver cells (Giobbe *et al.*, 2015; Danoy *et al.*, 2019). In our previous work, an approach based on the culture in microfluidic biochips has been introduced (Danoy *et al.*, 2019). Those protocols led to the improvement of the maturation of hiPSCs-derived HLCs as shown by the increase in the production of albumin and in the functionality of CYP450 when compared to conventional protocols. Additionally, the apparition of endothelial-like cells was observed, but advanced endothelial hepatic markers such as Stabilin-2 could not be detected.

To tackle the remaining issues in monocultures of HLCs, we propose in this manuscript an extension of our investigations by performing cocultures of hiPSCs-derived LSECs and HLCs in a fluidic microenvironment. As cocultures have been shown to impact the differentiation and the maturation of primary hepatocytes, the presented cultures are expected to have an impact on the processes of differentiation and regulation of the inflammatory phenomena.

## II. RESULTS

### A. Cell morphology

**FIG. 1.**
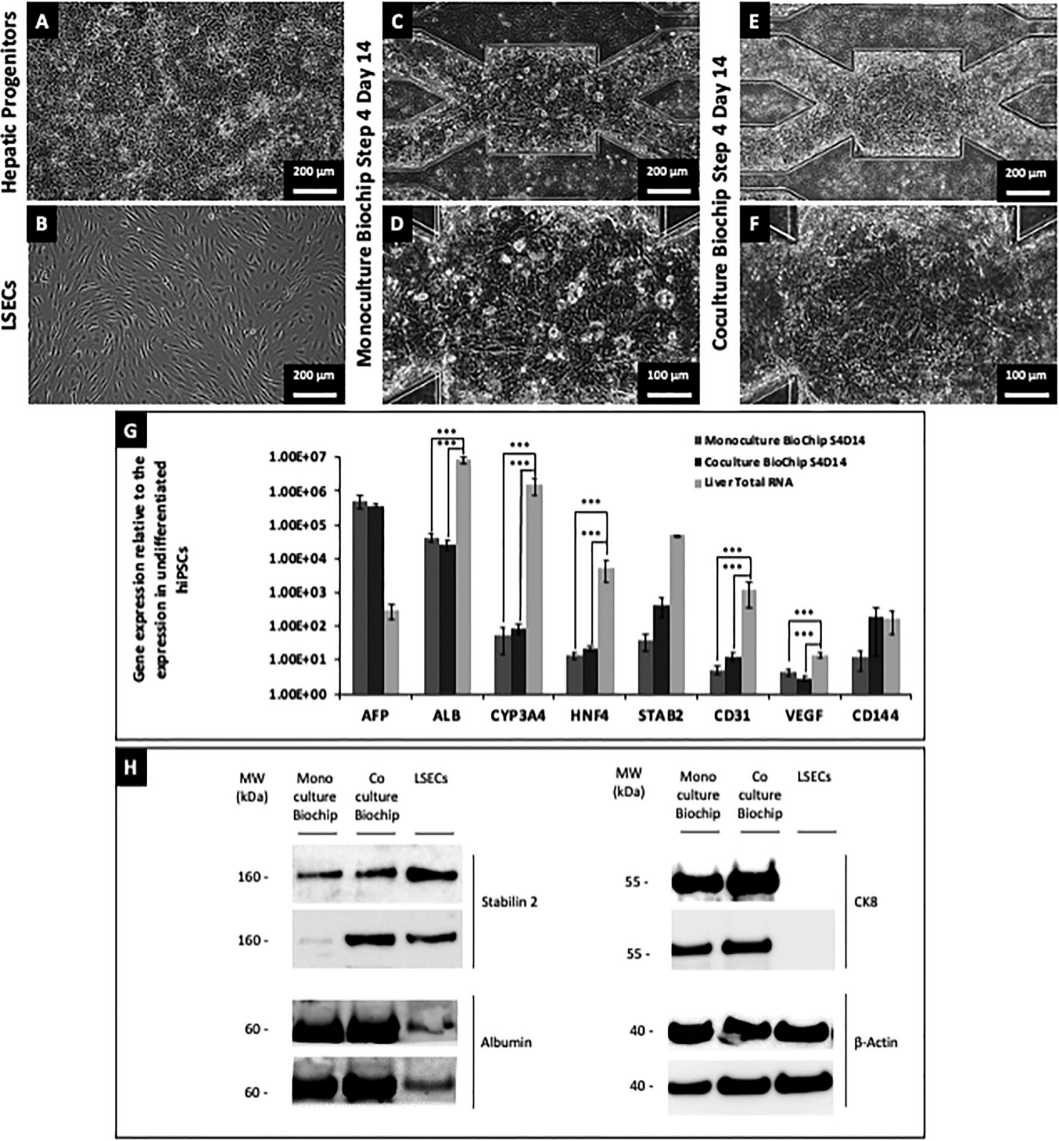
Cell morphology of hiPSCs-derived hepatic progenitors (a) and LSECs (b) before their detachment and insertion in biochips. Morphologies observed in monoculture [(c) and (d)] and coculture [(e) and (f)] biochips after 14 days of maturation in step 4. Gene expression levels of AFP, ALB, CYP3A4, HNF4, STAB2, CD31, VEGF, and CD144 measured by RT-qPCR (g) for Liver Total RNA, monoculture, and coculture biochips. Data are presented as mean ± SE. Duplicate of total protein extracts from hiPSCs-derived HLCs controls in Petri dishes, monoculture biochips, coculture biochips, hiPSCs-derived LSECs controls analyzed by western blots for Stabilin-2, albumin, CK8, and beta-actin (H). Full images of western blots are given in supplementary material File 1, Figs. S3 and S4.

hiPSCs-derived hepatic progenitors and LSECs exhibited typical morphologies before harvesting [Figs. 1(a) and 1(b)]. The former presented a tissue composed of a monolayer of cells with randomly distributed aggregates as previously observed (Danoy *et al.*, 2019). After insertion in biochips and further in the maturation, no specific difference could be observed in the tissue between monoculture and coculture biochips (Fig. S2 in supplementary material File 1). After 14 days of maturation in monoculture biochips [Figs. 1(c) and 1(d)] and coculture biochips [Figs. 1(e) and 1(f)], no specific difference was further observed in the morphology of the tissue. In both conditions, the tissue was found to be thicker in proximity to the microstructures. Within the center, the microchambers and microchannels, large cuboid cells with a large nucleus could be observed. In proximity to the microstructures, cell morphology could not be distinguished, indicating a thicker fibroblastic tissue that was found to reach the top of channels.

### B. RT-qPCR

After 14 days of maturation in biochips, no statistical difference was found at the gene level between coculture and monoculture biochips for the investigated genes [Fig. 1(g)]. Especially, endothelial markers such as STAB2, CD31, VEGF, and CD144 were detected in both monoculture and coculture biochips. High variation of the expression of those markers in monoculture biochips led to the lack of statistical significance in the comparison with coculture biochips. Markers such as ALB, CYP3A4, HNF4, CD31, and VEGF were found to be statistically higher in samples extracted from Liver Total RNA than in monoculture and coculture biochips.

### C. Western blot

Detection of proteins by western blot confirmed the presence of Stabilin-2 in hiPSCs-derived LSECs and coculture biochips [Fig. 1(h)]. A basal expression of Stabilin-2 was also detected in few monoculture biochips as shown in selected replicates. Concerning hepatic markers, albumin and CK-8 were detected in both monoculture and coculture biochips. The expression of albumin in hiPSCs-derived LSECs was considered non-significant as it was found to be lower than in undifferentiated hiPSCs (Figs. S3 and S4 in supplementary material File 1). While the detection was only performed on a single sample due to antibody crosstalk, FOXA2 and HNF4 were not found to be expressed in any condition, but CYP3A4 was interestingly found to be only expressed in coculture biochips (Figs. S3 and S4 in supplementary material File 1).

### D. Immunohistochemistry

**FIG. 2.**
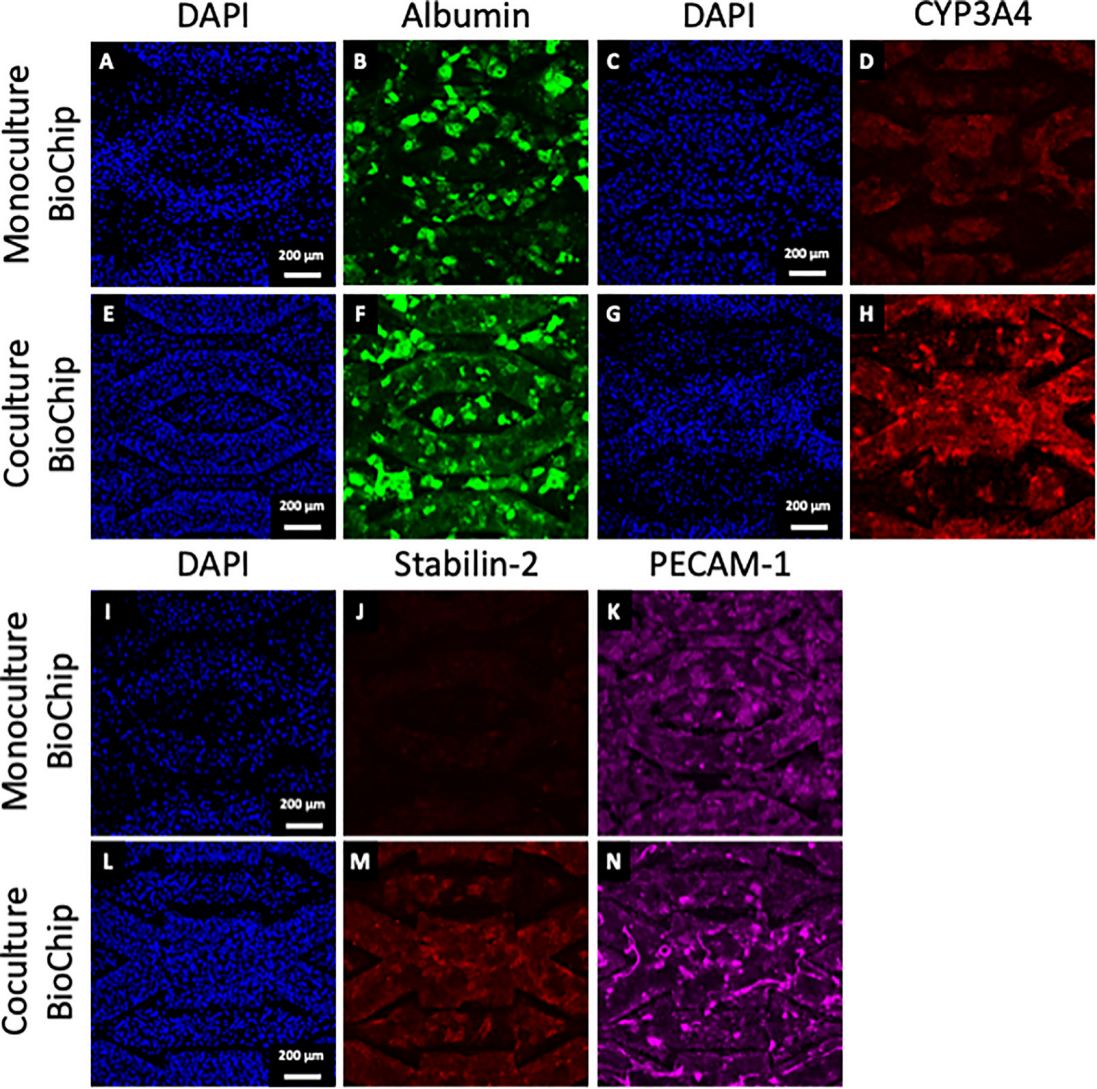
Counterstaining of DAPI with corresponding fluorescent immunostaining images for albumin and counterstaining of DAPI with corresponding fluorescent immunostaining images for CYP3A4 in monoculture biochips [(a)–(d)] and coculture biochips [(e)–(h)] after 14 days of maturation. Counterstaining of DAPI with corresponding fluorescent immunostaining images for Stabilin-2 and PECAM-1 in monoculture biochips [(i)–(k)] and coculture biochips [(l)–(n)] after 14 days of maturation.

Expression of albumin was confirmed in both monoculture and coculture biochips after 14 days of maturation, while the expression of CYP3A4 was found to be higher in coculture biochips [Figs. 2(a)–2(h)]. Stabilin-2 was found to be widely expressed in coculture biochips, while PECAM-1 was found to be expressed in both monoculture and coculture biochips [Figs. 2(i)–2(n)]. Stabilin-2 was not found to be expressed in monoculture biochips. Interestingly, a tubular-like network, positive to PECAM-1, was observed in coculture but not in monoculture biochips.

### E. Functional assays

**FIG. 3.**
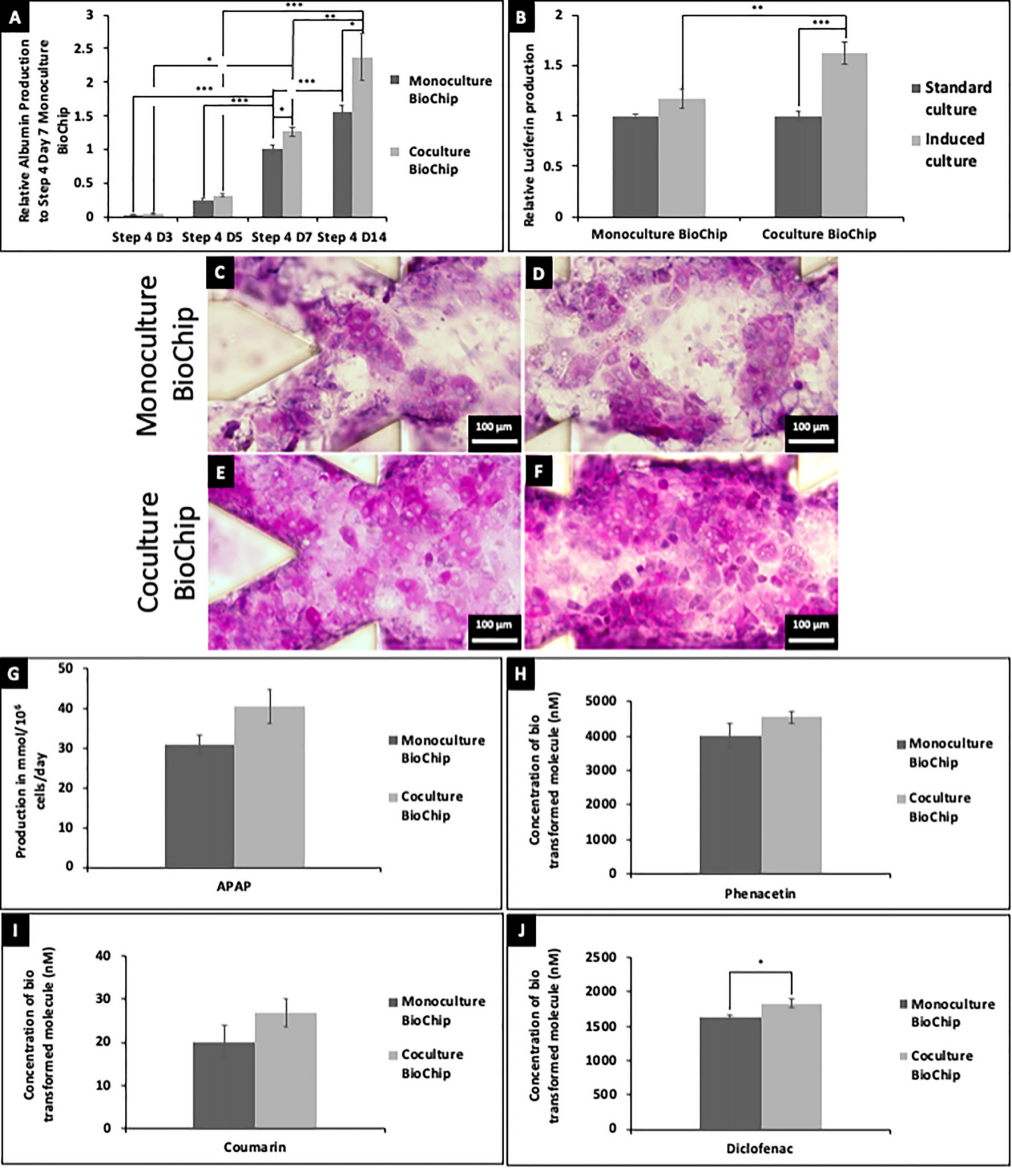
Albumin production in monoculture and coculture biochips measured by ELISA (a). Luciferin production resulting from the CYP3A4 activity assay performed in monoculture and coculture biochips standard and induced cultures (b). PAS staining in different areas of monoculture [(c) and (d)] and coculture biochips [(e) and (f)] after 14 days of maturation. Metabolites produced in monoculture and coculture biochips through the exposition of cells to APAP (g), phenacetin (h), coumarin (i), and diclofenac (j) present in the drug cocktail. Data are presented as mean ± SE.

Levels of albumin were found to be increasing throughout the maturation in biochips [Fig. 3(a)]. Notably, after 7 days and 14 days of maturation in biochips, the albumin levels were found to be statistically higher in coculture (on average, 5.31 *μ*g/mL/10^6^ cells) than in monoculture biochips (in average, 3.19 *μ*g/mL/10^6^ cells). The luciferin-Ingenuity Pathway Analysis (IPA)-based CYP3A4 assay revealed comparable levels in monoculture and coculture biochips [Fig. 3(b)]. However, induction of the tissue was only found to be significant in coculture biochips after 14 days of maturation. The capacity of the tissue to store glycogen was found to be even in biochips and higher in coculture than in monoculture biochips as shown by Periodic Acid-Schiff (PAS) staining [Figs. 3(c)–3(f)]. Drug assays were performed on non-induced biochips in perfusion for 24 h. No significant difference was detected in the metabolism of N-acetyl-para-aminophenol (APAP) [Fig. 3(g)], phenacetin [CYP1A2, Fig. 3(h)], and coumarin [CYP2A6, Fig. 3(i)]. Higher metabolism of diclofenac was detected in coculture biochips [CYP2C9, Fig. 3(J)]. Due to the high adsorption of bupropion, bufuralol, midazolam, and amodiaquine in the microfluidic circuit, no measurable values could be detected.

### F. Staining of collagen and angiogenesis array

**FIG. 4.**
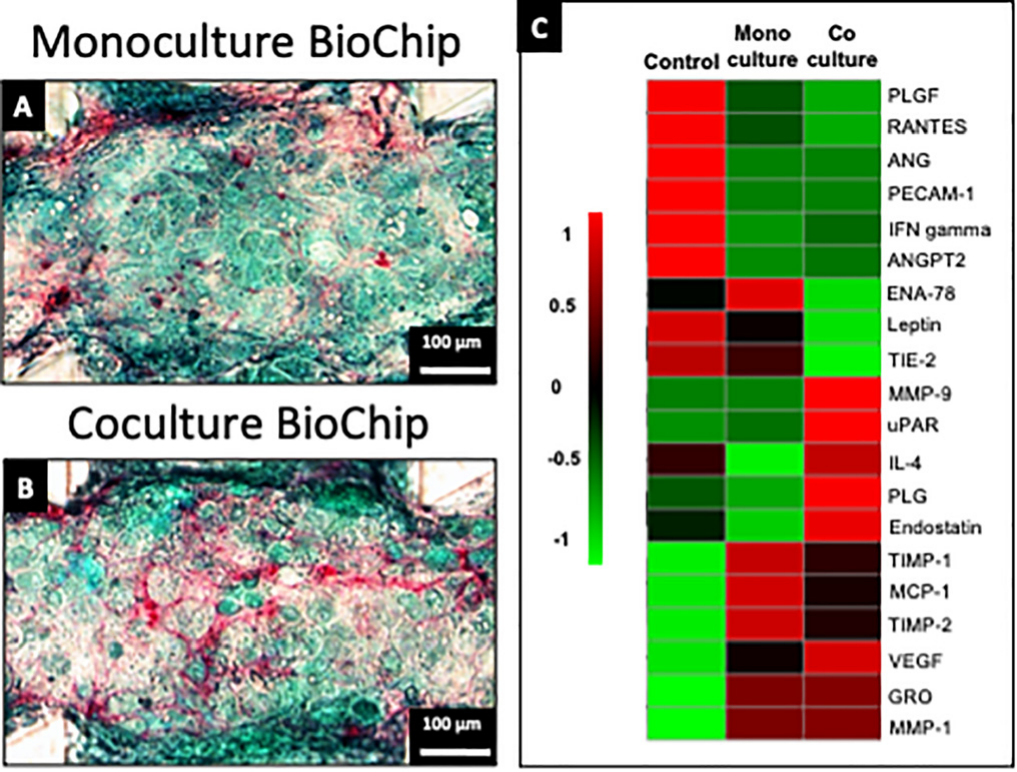
Sirius Red and Fast Green staining of monoculture (a) and coculture biochips (b) after 14 days of maturation. Cytokines and pro-angiogenic factors measured in monoculture and coculture biochips, and in the culture medium control (c, detailed results in Table S4).

Sirius Red staining revealed the presence of a dense collagen network in both monoculture and coculture biochips [Figs. 4(a) and 4(b)]. In monoculture, staining of fibers in red was found to occur mainly along the microchannels and microchamber walls. In coculture, the collagen network appeared more developed and positioned in all parts of the microchannels, including in their center. Statistical analysis on the proteins from the angiogenesis array indicated higher levels of VEGF, PLG, Endostatin, iL-4, MMP9, and uPAR in coculture biochips, while ENA-78, Leptin, MCP1, TIMP1, and TIMP2 were found to be higher in monoculture biochips [Fig. 4(c)]. By doing a comparison with the control culture medium, ANG, IFN-gamma, PLGF, RANTES, ANGPT2, and PECAM-1 were found to be consumed in both monoculture and coculture biochips, while GRO and MMP1 were found to be produced. The measured values for all proteins from the angiogenesis array are given in Table S4 in supplementary material File 1.

### G. Transcriptome analysis

**FIG. 5.**
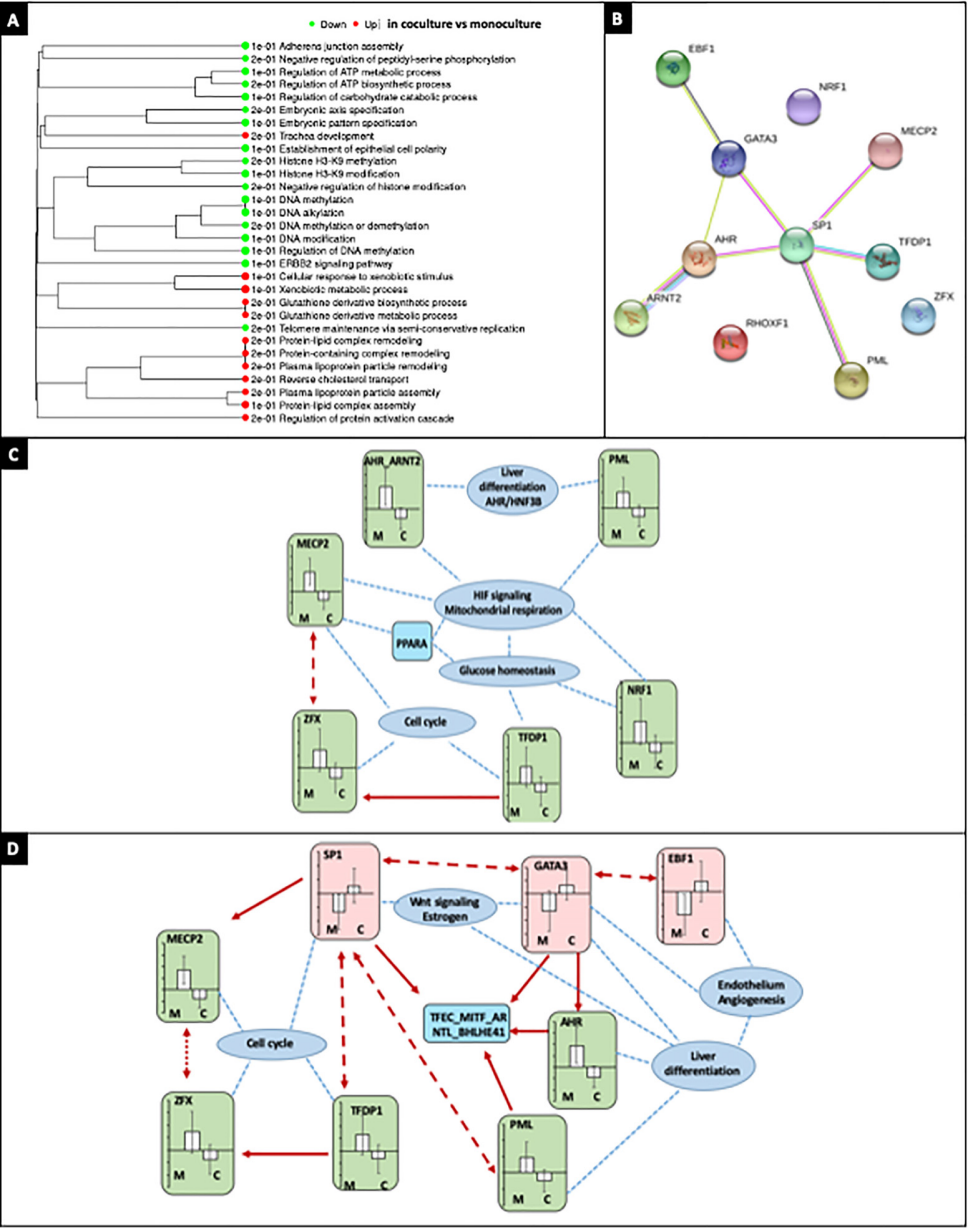
Differentially expressed pathways in monoculture and coculture biochips as extracted by the iDEP GSEA analysis (a). Connection between the top-10 differentially activated TFs in monoculture and coculture biochips identified by ISMARA and obtained by STRING processing (b). First interaction network formed with the TF motifs highlighted by MARA analysis in the comparison between monoculture and coculture biochips, and their target pathways (c). Second interaction network formed with the TF motifs highlighted by MARA analysis in the comparison between monoculture and coculture biochips, and their target pathways (d).

The comparison of the transcriptomic profiles of monoculture and coculture biochips after 14 days of maturation in biochips performed with iDEP (False discovery rate of 0.1, fold change of 2) did not lead to significant discrimination between the two conditions in the DESQ2 analysis. However, using pathway analysis, a clear distinction between the two culture conditions could be made. The Gene Set Enrichment Analysis (GSEA) formalism led to the extraction of several GO_biological processes (xenobiotic metabolism, glutathione processing, cholesterol, and various lipids regulation), upregulated in coculture biochips. In monoculture biochips, adenosine triphosphate (ATP) and DNA processing was found to be upregulated [Fig. 5(a), the complete dataset is given in supplementary material File 2]. The GO_KEGG analysis (Table I) led to the extraction of several pathways (related to drug and CYP450 metabolism and fluid shear stress), upregulated in coculture biochips.

**TABLE I.** KEGG pathway upregulated in coculture biochips when compared to monoculture biochips with the iDEP pathway analysis.

Pathway	Gene number	Adj. *P* value
Chemical carcinogenesis	40	3.5 × 10^−3^
Drug metabolism	35	3.5 × 10^−3^
Complement and coagulation cascades	50	3.5 × 10^−3^
Metabolism of xenobiotics by cytochrome P450	38	6.5 × 10^−3^
Retinol metabolism	34	4.8 × 10^−2^
Glutathione metabolism	40	4.8 × 10^−2^
Drug metabolism	52	1.0 × 10^−1^
Fluid shear stress and atherosclerosis	112	1.0 × 10^−1^

### H. Motif Activity Response Analysis

The transcription factors (TFs) differentially expressed between monoculture and coculture biochips were extracted by ISMARA. From the analysis, TFs with the highest activity in monoculture (MECP2, AHR_ARNT2, PML, NRF1, ZFX, and TFDP1) and coculture biochips (SP1, EBF1, RHOXF1, and GATA3) were identified (Table II). A network bridging those TFs could be established by SPRING processing [Fig. 5(b)]. From the top target analysis in ISMARA, the regulatory networks between TFs, targeted molecules, and the related downstream pathways could be established [Figs. 5(c) and 5(d), complete dataset in supplementary material File 3]. In monoculture, the central mechanisms highlighted were glucose homeostasis, hypoxia-inducible factor (HIF) signaling, and processes related to the cell cycle and liver differentiation. By adding the TFs with higher activity in coculture biochips (SP1, GATA3, and EBF1), mechanisms related to angiogenesis and the endothelium could be highlighted. Additionally, mechanisms related to the Wnt signaling and estrogen signaling could also be highlighted. In monoculture, PPARA was found to be a potential central node, while the TFEC_MITF_ARNTL_BHLHE41 motif was found to be a potential central node in the coculture biochips.

**TABLE II.** Top 10 TFs motifs extracted in monoculture biochips (M) and coculture biochips (C) biochips by ISMARA and sorted by z-value. “TFs motif” represents a set of transcription factors with similar target nucleotide sequence pattern identified by ISMARA. “PCC” is the gene with strongest Pearson correlation coefficient for the TF motif identified. “Pattern” describes the nucleotide sequence pattern of the motif for the PCC selected genes. The details of the analysis are given in supplementary material File 3.

TFs motif	Z-value	PCC	Up	Pattern	Potential liver interest
MECP2	1.15	MECP2	M	CCCGGAG	NCadherin, HIF signalings
					Cell cycle
					PPARA
AHR_ARNT2	0.98	AHR_ARNT2	M	TGCGTG	IL6, STAT3 pathways
					Hypoxia, xenobiotics responses
SP4_PML	0.9	PML	M	GGGGCCAGGGGGGGGGCGGGGCCG	HIF, HNF3B pathways
					ERK/MAPK signaling
					VEGF ligand
SP1	0.87	SP1	C	GGGGGCGGGGC	Actin processing
					RAC1, Wnt signalings
					Cell cycle
EBF1	0.87	EBF1	C	ACCCAAGGGA	Lymphopoiesis
					Glomerular endothelium
RHOXF1	0.81	RHOXF1	C	ATAATCCC	
NRF1	0.77	NRF1	M	CTGCGCATGCGC	Mitochondrial respiration
					Glucokinase
ZFX	0.75	ZFX	M	GGGGCCGAGGCCTG	Cell cycle
GATA3	0.72	GATA3	C	AGATGG	Liver ESC differentiation
					Angiongenesis regulation
					Wnt pathway
TFDP1	0.71	TFDP1	M	GGCGCG	G1/S phase
					JAK/STAT
					Regulation of glucose

### I. Proteome analysis

**FIG. 6.**
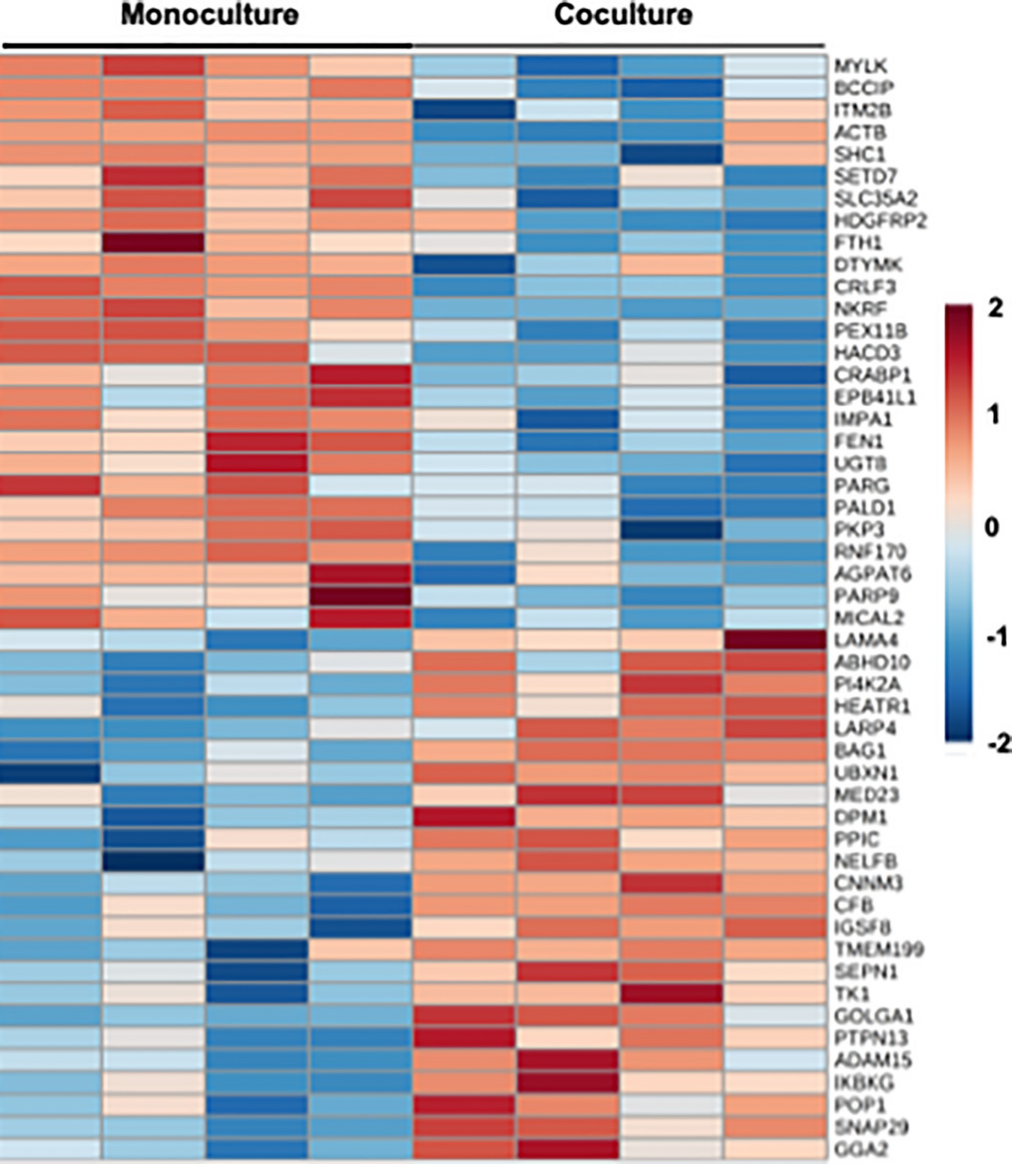
Heatmap recapitulating the differentially expressed proteins in monoculture and coculture biochips (detailed results in supplementary material File 4).

Partial Least Squares Discriminant Analysis (PLS-DA) on the proteomic dataset allowed us to discriminate monoculture from coculture biochips (Fig. S5 in supplementary material File 1). However, as far as the value of the cross-validated predictive ability (Q^2^) is concerned, the predictability of the model remained poor. Nonetheless, statistical analysis has allowed extracting 104 proteins differentially expressed between monoculture and coculture biochips (*P* value below 0.05, Fig. 6, the complete dataset is given in supplementary material File 4). In monoculture biochips, *NFKB1*, *NKRF*, *MALT1* (NFKB pathway), *ESAM* (endothelial adhesion molecule), *SHC1* (response to ROS, regulation of angiogenesis and sprouting, regulation of lipid metabolism), *MILCA2* (response to VEGF), and ECM-related proteins (*MILCA2, MYLK, ACTB, PKP3*) were found to be over-expressed. In coculture biochips, *ANGPT2* (angiogenesis), cell cycle proteins (*CHEK1*, *CDK4*, *CDK9*), *CFB* (complement factor B), *SNCA* (expressed in vascular endothelial cells), and some heat shock protein linked to chaperone proteins *HSPA*, *HSPB6*, *DNAJB12*) were found to be overexpressed.

By inputting those proteins in the Ingenuity Pathway Analysis (IPA), several canonical pathways linked to inflammation, oxidative stress, and acute phase response were highlighted. Additionally, *APP*, *GFPT1*, *BMI1*, *ERBB2*, and *ERBB3* were proposed as upstream regulators (Table S5 in supplementary material File 1). By analyzing the KEGG pathways, the enrichment of pathways related to inflammation and the cell cycle could be extracted. Moreover, proteins related to peroxisome, phospholipase, and lipid metabolism (*HACD3*, *PEX11B*, *UGT8*, *RRAS2 in monocultures*, *FAR1*, *FADS3*, *GOLGA1*, *in cocultures*), and cellular remolding and reorganization (*LAMA4* overexpressed in cocultures*, ATCB, MYLK* overexpressed in monocultures) were also extracted. The complete list of pathways extracted is given in supplementary material File 4.

### J. Metabolome analysis

**FIG. 7.**
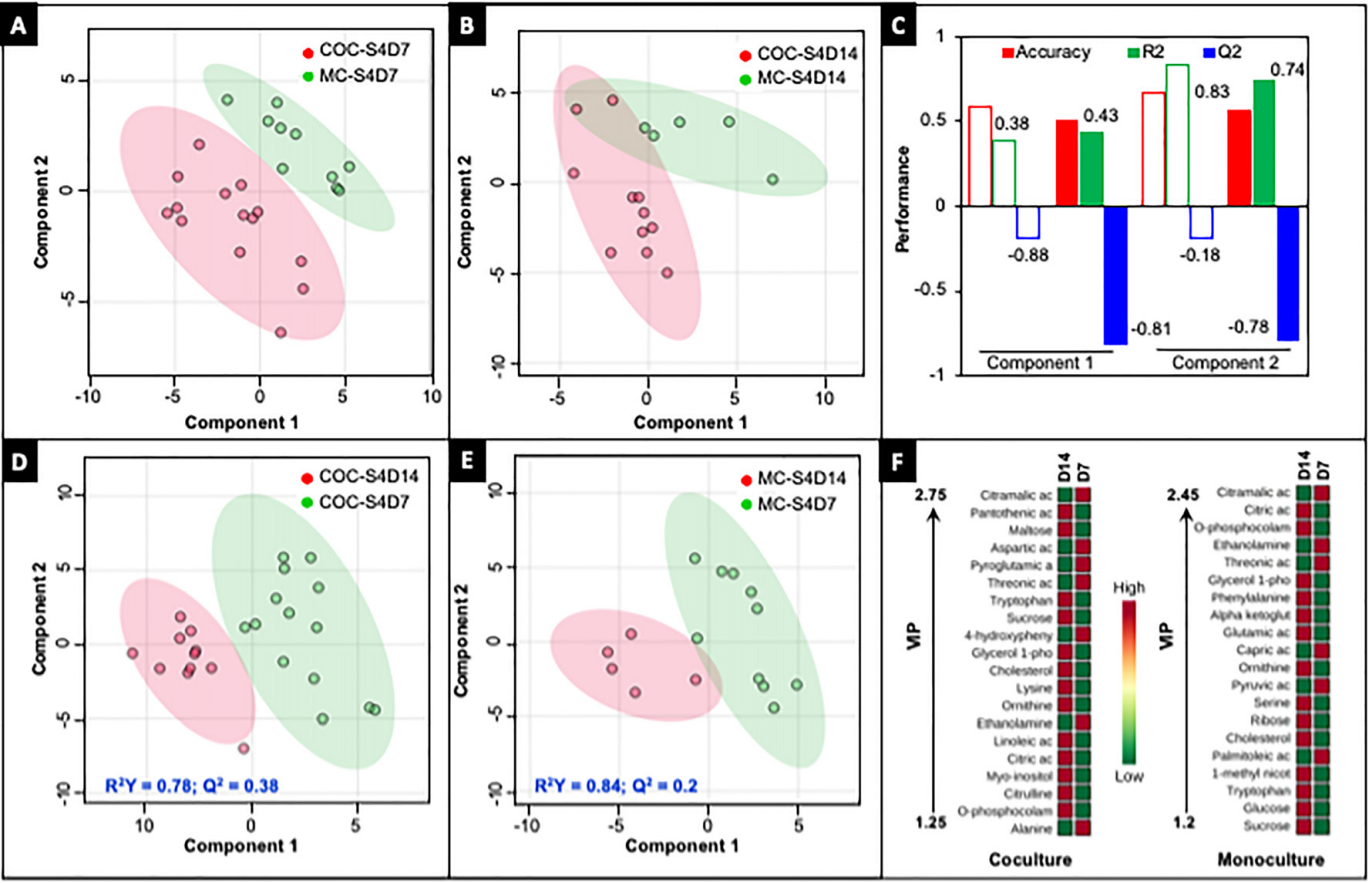
Multivariate statistical analysis based on the GC-MS profiles in monoculture and cocultures biochips after 7 and 14 of maturation. PLS-DA scores plot discriminating both culture modes after 7 (a) and 14 days (b) of maturation. PLS-DA cross-validation. Performance parameters (accuracy, R^2^, and Q^2^) assessed using different components. [Empty bars obtained from PLS-DA (a); Solid bars obtained from PLS-DA (b)] (c). PLS-DA scores plot discriminating 7 and 14 days of maturation in coculture (d) and monoculture biochips (e). Top 20 metabolites differentially expressed (VIP > 1) after 7 and 14 days of maturation in both monoculture and coculture biochips (f).

Analysis of the metabolome was performed in both monoculture and coculture biochips after 7 and 14 days of maturation. The Gas chromatography coupled to mass spectrometry (GC-MS) analysis led to the identification of 86 metabolites differentially expressed between the four conditions. A multivariate analysis on those allowed us to identify the specific signatures for each condition. Normalization of the datasets to a blank culture medium was performed to allow for comparison. While Principal Component Analysis (PCA) was not able to discriminate the different conditions, supervised PLS-DA showed a better separation [Figs. 7(a) and 7(b)] but with a low predictive ability as illustrated by the Q^2^ values [Fig. 7(c)]. Only three components (ribose, oleic, and palmitoleic acids) were found to be statistically different in monoculture and coculture biochips after 7 days of maturation, while only one component (triethanolamine) was found to be statistically different in monoculture and coculture biochips after 14 days of maturation (*P* value below 0.05, supplementary material File 5). Comparison between 7 days and 14 days of maturation in coculture biochips [Fig. 7(d) Q^2^ = 0.38] led to the extraction of 15 metabolites differentially expressed in either condition (*P* value below 0.05, VIP > 1). In detail, higher levels of citramalic, aspartic, pyroglutamic, threonic and 4-hydroxyphenylacetic acids, and ethanolamine were found after 7 days of maturation, while higher levels of pantothenic acid, maltose, tryptophan, sucrose, trans-4-hydroxy-l-proline, glycerol 1-phosphate, cholesterol, lysine, ornithine, and linoleic acid were found after 14 days of maturation (supplementary material File 5). Comparison between 7 days and 14 days of maturation in monoculture biochips [Fig. 7(e), Q^2^ = 0.2] led to the extraction of nine metabolites differentially expressed in either condition (*P* value below 0.05, VIP > 1). In detail, the differentially expressed metabolites were citric, citramalic, threonic, glutamic, and alpha-ketoglutaric acids, O-phosphocolamine, ethanolamine, phenylalanine, and glycerol-1-phosphate (supplementary material File 5). Finally, a comparison of the top 20 VIP metabolites in each culture condition was performed [Fig. 7(f)]. In those, 10 metabolites were found to be common, illustrating the relatively close metabolic profiles of monoculture and coculture biochips.

## III. DISCUSSION

In this manuscript, the coculture of hiPSCs-derived HLCs and LSECs was performed in a fluidic microenvironment. Investigation of the levels of differentiation and maturation of the cells was performed at a transcriptomic, proteomic and metabolomic level. The presence of both cell types was confirmed by RT-PCR, western blot, and immunostaining [Figs. 1(g), 1(h), and 2). Immunostaining has allowed locating both HLCs and LSECs-derived hiPSCs as evenly distributed in all areas of the biochips. Improvement of the maturity of the tissue was notably found at a functional level, especially in the production of albumin and in the drug metabolism (Fig. 3). In detail, the albumin production (10.62 *μ*g/day/10^6^ cells) reached levels higher than those of fetal human hepatocytes or HepG2 cultured in similar microfluidic biochips (47 ng/day/10^6^ cells and 2.5 *μ*g/day/10^6^ cells, respectively, Leclerc *et al.*, 2004) and within the range observed in human primary hepatocytes cultured in those biochips (9.6 *μ*g/day/10^6^ cells, Jellali *et al.*, 2016). It is also important to note that the albumin production rate remains much lower than the *in vivo* estimation (35–105 *μ*g/day/10^6^, Baudy *et al.*, 2020). More interestingly, drug metabolism and CYP450 activity were confirmed in those experiments which consist of a clear improvement to previous results (Danoy *et al.*, 2019). The results presented are also consistent with the literature on coculture of primary hepatocytes and LSECs for the improvement of hepatic maturation and its maintenance (Bale *et al.*, 2015).

Previous reports in our group have shown that the maturation of hiPSCs-derived HLCs in biochips led to the formation of multi-cellular tissue (Danoy *et al.*, 2019). Indeed, as cells are injected in biochips at a progenitor stage, they still retain part of their pluripotency which allows them to differentiate toward a different lineage in the biochip. Especially, the presence of endothelial-like cells and hypoxia-related phenotypes was confirmed in longer biochips after 7 days of maturation (Danoy *et al.*, 2019, Danoy *et al.*, 2020b). In the current setup, the length of the biochip was reduced by 5 folds and hypoxia-related phenotype was not highlighted. As in monoculture, the observed endothelial-like cells expressed few specific LSECs markers, and the inclusion of relevant LSECs models such as the one previously described (Koui *et al.*, 2017) was performed in the present study. By doing so, the coculture of both hiPSCs-derived HLCs and LSECs was confirmed in the biochip. Interestingly, the presence of a complex PECAM-1 positive tubular network in the middle of the microchannels and a larger expression of Stabilin-2 in the tissue was found [Fig. 2(n)]. However, as PECAM-1 is known to disappear in LSECs during the sinusoid formation in the lobule (Poisson *et al.*, 2017), its expression in the coculture biochips could be reflective of an ongoing sinusoid formation, exhibiting an immature profile. Nonetheless, coculture biochips also exhibited important angiogenic signaling [VEGF, ANGPT2, Fig. 4(c), Biel and Siemann, 2016] which highlights the important crosstalk between the cell types present in the biochip.

Similitudes in the transcriptomic profile of both coculture and monoculture biochips were found. This could be explained by the derivation of part of the cells produced by the HLCs differentiation protocol into nonspecific endothelial cells (Danoy *et al.*, 2019) which influences the general profile of monoculture biochips. The inoculation of sorted hepatoblasts in the biochips or analysis on cell population sorted at the end of the experiment might partly solve this issue. Nevertheless, differences were found in coculture and monoculture biochips as far as specific upstream TFs were over-expressed in the former. Among those, links between liver differentiation and patterns related to the endothelium could be made through EBF1 and GATA3 [Fig. 5(d)]. GATA3 is known to be involved in endothelial differentiation (Song *et al.*, 2009; Lagarkova *et al.*, 2008) and to be a key factor in the specification of stem cells toward hepatic phenotypes (Yamamizu *et al.*, 2013). EBF1 has been hypothesized to play a role in the differentiation of stem cells into cholangiocytes and hepatocytes (Armartmuntree *et al.*, 2018) but more importantly in the differentiation of mesenchymal tissues (Almalki and Agrawal, 2016) and the endothelial development in kidneys (Brunskill and Potter, 2010). In the present work, both of those TFs could potentially be linked to the development of the tubular structure observed in the biochip.

The metabolomic profiles of coculture and monoculture biochips after 14 days of maturation were also found to be comparable. However, differences were more pronounced in the comparison of 7 and 14 days of maturation in coculture biochips. Indeed, higher concentrations of lipids were found after 14 days of maturation which is consistent with the genomic analysis in which PPARA and related pathways were linked by the TFs activity [Fig. 5(d)]. Those results could be related to previously observed fatty liver patterns (Lee *et al.*, 2015) and are consistent with *in vivo* regenerative patterns in the liver (Rudnick and Davidson, 2012). In the present work, higher expression of FADS3 was found in the proteomic dataset of coculture biochips. This could be linked to a higher capability of desaturation of intracellular fatty acids which may illustrate better oxygenation of the tissue and which is consistent with the development of endothelial tissue.

Staining in biochips revealed the presence of a dense collagen network [Figs. 4(a) and 4(b)]. In coculture, the staining appeared located in the center of the channels in proximity to the PECAM-1 tubular structures detected by immunostaining [Fig. 2(n)]. Liver regeneration and angiogenesis have been frequently linked (Poisson *et al.*, 2017; Gressner *et al.*, 2002; Elpek, 2015). Moreover, VEGF, known as an important regulator of angiogenesis, is also known to be involved in fibrogenesis and in the release of angiocrine signals by LSECs which control liver regeneration and fibrogenesis signaling (Poisson *et al.*, 2017). In the present dataset, higher levels of VEGF, ANGPT2, Endostatin, VEGFR2, VEGFR3, and TIE2 [Fig. 4(c)] were found in coculture biochips and describe well the closely related signature of angiogenesis and liver regeneration observed in the culture.

As signaling related to liver regeneration and angiogenesis are often linked with inflammatory signaling, a pro-inflammatory-like response could be expected in biochips. Initially, inflammation markers were found to be overexpressed in monocultures [TIMP1, TIMP2, MCP1, ENA78, Fig. 4(c); *NFKB*, *MALT1* proteins in supplementary material file 4] as compared to coculture. Additionally, the observed activations of NFKB, STAT3, and PI3K have been confirmed to be strongly linked to events observed in the case of liver regeneration events caused by hepatectomy (Michalopoulos, 2010). Moreover, hepatic proliferation has been defined as the concerted action between MET and EGFR receptors with several cytokines including tumor necrosis factor (Michalopoulos, 2010). Finally, the termination of liver regeneration has been associated with the interactions between HGF, TGFB1, and extracellular matrix proteins (Apte *et al.*, 2009; Michalopoulos, 2010). In the present dataset, coculture biochips presented a high production of collagen (Fig. 4) which would be expectedly linked to a fibrotic-like response or a pro-inflammatory state. However, those events could not be linked so far as higher levels of ENA78, TMP1, and TMP2 were found in monoculture biochips and higher levels of anti-inflammatory markers such as IL-4 and MMP9 were found in coculture biochips. Besides, the proteomic dataset also exhibited higher levels of the NFKB signaling protein in monocultures which further corroborates those theories. Finally, the metabolomic analysis did not reveal signatures related to cellular stress. Production of pyroglutamate and modulation of glutamate and methionine were shown to be involved in the response to oxidative stress and liver toxicity (Liu *et al.*, 2014) and are often linked to inflammation which suggests that no patterns specific of inflammation were observed in coculture biochips when compared to monoculture.

## IV. CONCLUSION

In the present work, coculture of hiPSCs-derived LSECs and HLCs was shown to contribute to the improvement of the maturity (albumin production has reached 5.31 *μ*g/mL/10^6^ cells in coculture biochips) and of the function of the latter, notably regarding drug metabolism and its inducibility. hiPSCs-derived LSECs were also found to contribute to the formation of a tubular-like network within the tissue while collagen fibers were found in its proximity. While fibrotic patterns could be suspected, the multi-omics analysis has allowed revealing typical regenerative liver patterns which are often entangled with an angiogenetic signature. Further investigations, using technics such as cell sorting could help toward a better differentiation of patterns between monoculture and coculture biochips while the further study of the liver regenerative process in biochips appears as one of the possible applications of the organ on chip technology.

## V. METHODS

### A. Differentiation protocols

Differentiation of hiPSCs-derived HLCs was performed using the TKDN4-M clones (Institute of Medical Science, the University of Tokyo, Takayama *et al.*, 2010). Before induction, cells were seeded at a 10 000 cells/cm^2^ density on Matrigel-coated Tissue Culture Polystyrene (TCPS) dishes (20 mg/mL, Corning Matrigel hESC-Qualified Matrix). After proliferation, cells were allowed to follow a four-step differentiation process (referred to as step 1 to 4). The different culture conditions observed in those steps have been detailed in Table S1 (supplementary material File 1). VEGF was added in coculture conditions to favor the development of LSECs but was omitted in monocultures as it has been shown to not affect hepatocytes unless in the presence of LSECs (LeCouter *et al.*, 2003). Differentiation of hiPSCs-derived LSECs was performed using the 454E2 clones (Riken BioResource Research Center, RIKEN BRC). Those cells were handled as previously described (Koui *et al.*, 2017) and were characterized in previously published manuscripts (Danoy *et al.*, 2020a). Once differentiated, hiPSCs-derived LSECs were detached and stored in liquid nitrogen. After thawing, the cells were seeded at a 1.5 × 10^4^ cells/cm^2^ density on fibronectin (20 *μ*g/mL, 1 h, 37 °C, Life Technologies) coated TCPS plates. Cells were then further cultured and proliferated for an additional 9 days before being inserted in biochips.

### B. Maturation in biochips

The design, fabrication, and assembly processes of the microfluidic biochips were previously reported fully (Baudoin *et al.*, 2007; 2011). In detail, the biochip, made from two layers of PDMS (polydimethylsiloxane), consisted of a single culture chamber of 1.2 cm length, 1 cm width, and a total 300 *μ*m height. At the bottom of this chamber, an array of microchannels and microchambers was designed, and cells were allowed to adhere both in and on the microfabricated structure. Schematics and images of the device are given in Fig. S1 in supplementary material File 1.

Before using, biochips were sterilized with an autoclave and coated with a Matrigel solution (20 mg/mL, overnight, 37 °C), inserted with the help of 1 ml syringes. For seeding, hiPSCs-derived HLCs (between steps 3 and 4) and LSECs (after 9 days of proliferation) were harvested individually. In both monoculture and coculture biochips, a ratio of 9 cm^2^ of hiPSCs-derived HLCs per biochip was used. In addition to those, hiPSCs-derived LSECs were added with a 9 cm^2^/biochip ratio to coculture biochips. Cell seeding was then performed by pipetting directly in the tubing a volume of 40 *μ*L using a 200 *μ*L pipette tip. 1 ml syringes were then plugged onto the device, and the position of the cells was manually adjusted within the biochip. Upon cellular adhesion, the tissue was found to be uniform over the microchannels and microstructures. As controls, hiPSCs-derived HLCs and LSECs were also cultured in Petri dishes. Cellular adhesion and the formation of the tissue in biochips were favored by performing the culture in static conditions for 48 h after seeding. In this step, media was changed twice a day by using 1 ml syringes. Subsequently, perfusion was initiated at a 10 *μ*L/min flow rate which corresponds to a range of values of shear stress observed consistently in the literature and found to be suitable for the culture of hepatic cells (Baudoin *et al.*, 2014). After 14 days in total of culture in step 4, perfusion was halted, and further analysis was performed on the samples. Normalization of the number of cells was performed by counting the total number of cells at the end of the maturation period. In this experiment, all cultures were performed under controlled atmospheric conditions (37 °C, 20% O_2_, 5% CO_2_) in an incubator. Perfusion of the biochips was done through a circuit composed of a peristaltic pump serially and a bubble trap connected to the biochips. To limit the adsorption of chemicals and growth factors, PFTE perfusion pipes were selected. Including biochips and bubble traps, the total volume of the perfusion circuit was found to be 2 ml.

### C. RT-qPCR

Protocols have been detailed in supplementary material File 1, and the primer sequences used in this study are listed in Table S2 (supplementary material File 1). The reference gene used was ACTB (β-Actin), and normalization of gene expression data was done by using undifferentiated hiPSCs as a reference sample.

### D. Western blots

Western blots were performed following the previously described protocol (Kopec *et al.*, 2017, detailed in supplementary material File 1). The antibodies used in this study were HNF4 HRP-conjugated (rabbit, ab209473, abcam), FOXA2 HRP-conjugated (rabbit, ab193880, abcam), CK8 HRP-conjugated (rabbit, ab193094, abcam), albumin HRP-conjugated (goat, A80–129P, Bethyl), and CYP3A4 HRP-conjugated (mouse, sc-53850 HRP, Santa Cruz). Super-Signal^®^ West Dura Extended Duration Substrate (Thermo Scientific) and a LAS-3000 imaging system (Fujifilm) were used to visualize the protein-antibody complexes by chemiluminescence.

### E. Immunohistochemistry

Staining was performed following the protocol detailed in supplementary material File 1. The antibodies used in this study were goat anti-albumin (A80–129A, Bethyl), rabbit anti-CYP3A4 (ab135813, abcam), rabbit anti-Stabilin-2 (ab121893, abcam), mouse anti-PECAM-1 (ab24590, abcam), donkey anti-goat Alexa Fluor 488 (ab150129, abcam), donkey anti-rabbit Alexa Fluor 568 (A10042, Thermofisher), and donkey anti-mouse Alexa Fluor 647 (ab150107, abcam). Fluorescent images were captured using a confocal microscope (PowerIX70, Olympus).

### E. Albumin measurements

Sandwich ELISA assays were performed to quantify the albumin in cultures. The antibodies used were an anti-human Albumin IgG (Bethyl, Japan, capture antibody) and an anti-human Albumin IgG coupled with peroxidase (Bethyl, Japan, detection antibody). The plate was read at 490 nm using the iMark Microplate reader (Bio-Rad) after peroxidase revelation by H_2_O_2_/OPD mixture.

### F. Periodic Acid Schiff staining

PAS staining was performed using a PAS staining kit (Muto Chemicals) following the manufacturers' recommendations. Before staining, samples were fixed 4% paraformaldehyde overnight at 4 °C before being washed with phosphate-buffered saline (PBS) for storage.

### G. Drug screening and CYP3A4 assay

A cocktail-substrate approach was used to evaluate the metabolic activity of the cells (Kozakai *et al.*, 2013). Components of the drug cocktail, as well as details of the analysis, are given in Table S3 (supplementary material File 1) and supplementary material File 1. The cocktail was diluted in culture medium and incubated for the last 24 h of culture. The possibility to induce metabolic activity in the tissue was investigated by incubation with inducers (5 *μ*M of rifampicin and 2 *μ*M of 3-methylcholanthrene). Drug metabolism was calculated considering fluidic adsorption as follows:

$$M\;=\;\left(Q_{\text{na}}-{\;Q}_{d}\right).$$ (1)

In which M is the quantity of drug biotransformed by the tissue, Q_na_ is the non-adsorbed drug quantity (control measured in a perfused circuit and an empty biochip), and Q_d_ is the remaining drug quantity in the perfusion loop after the assay.

### H. Sirius Red staining

Staining was performed using a Sirius Red/Fast Green Collagen Staining Kit (9046, Chondrex) following the manufacturer's recommendations.

### I. Angiogenesis array

Angiogenesis-related proteins were measured using a Human Angiogenesis Antibody Array (ab193655, abcam). Controls were performed using blank media from the coculture biochips. Measurements were made using a poll of six samples from each independent experiment (N = 3, n = 6) and with two technical replicates as recommended by the manufacturer.

### J. CAGE transcriptome profiling

The generation and sequencing of the NanoCAGE libraries were performed as previously described (Poulain *et al.*, 2017). To reduce the number of sequencing reads mapping to ribosomal DNA, pseudo-random primers were used (Arnaud *et al.*, 2016). Detailed protocols for the total RNA extractions, library preparation steps, sequencing, and data processing are given in supplementary material File 1. Custom R scripts using the CAGEr package were used to produce the expression tables uploaded on the iDEP server (http://ge-lab.org:3838/idep/) for differential gene expression and pathway analysis (Haberle *et al.*, 2015; Ge 2017).

### K. Proteomic analysis

Digestion of protein samples was performed, and separation of the peptides was done into five fractions. Drying by speed-vacuum was made, and the fractions were solubilized in 10 *μ*L of a 0.1% TFA, 10% Ceric Ammonium Acid (CAN) solution. A U3000 RSLC nanoflow-high-performance liquid chromatography (HPLC) system coupled to an Orbitrap fusion MS analysis (Thermo Fisher Scientific) was used for liquid chromatography and mass spectrometry analyses. A concatenation of human sequences from the Uniprot-Swissprot database (Uniprot, release 2018–06) and an incremented list of contaminants were used as a database for this study. On both peptides and proteins, the false discovery rate (FDR) was kept below 1%. Unique and razor peptides were used for the Label-free protein quantification (LFQ) and at least 2 ratio counts were required for the latter. Simultaneous analysis of all samples was performed with the “match between runs” option, with a match time window of 0.7 min and an alignment time window of 20 min. The protocol used in this study is fully detailed in supplementary material File 1. Multivariate statistical analysis was performed (PLS-DA, partial least squares-discriminate analysis), and Student's t-test (XLSTAT.2016, Addinsoft) was performed to analyze the proteomic dataset. Differences with a P-value of 0.05 or less were highlighted and considered significant. MetaboAnalyst 4.0 (Chong *et al.*, 2018) was used to highlight the top 50 differentially expressed proteins and to summarize them into a heatmap.

### L. Metabolomic analysis

Gas chromatography (Agilent 7890B) coupled to mass spectrometry (Agilent 5977A, GC-MS) was used to perform the metabolomic analysis on the culture medium from the different culture conditions. Rxi-5SilMS columns (30 m with a 10 m Integra-Guard column, 13623–127, Restek) were used. Preparation of samples and extraction of metabolites (Jellali *et al.*, 2018), GC-MS injections, and analysis steps (Fiehn *et al.*, 2008) were performed as previously described. The AMDIS software (http://chemdata.nist.gov/mass-spc/amdis/) was used for the analysis. Masshunter Quantitative Analysis (Agilent) was used to determine peak areas, and normalization was done to ribitol. Further details on the protocols for metabolite extraction and sample injection are given supplementary material File 1. The XLSTAT.2016 software (Addinsoft) and MetaboAnalyst 4.0 (Chong *et al.*, 2018) were used to perform the metabolomic multivariate data analysis. Significant variations between the groups were highlighted by unsupervised PCA and supervised PLS-DA. Differentially detected metabolites were identified according to their variable importance for the projection (VIP > 1) and P-value (Student's t-test, P < 0.05). MetaboAnalyst 4.0 (Chong *et al.*, 2018) was used to perform the metabolic pathway analysis.

### M. Statistical analysis

Data issued from the RT-qPCR, the drug screening, albumin measurements, and the angiogenesis array were analyzed using ANOVA (analysis of variance) to evaluate differences between the groups. When the null hypothesis was rejected, a *post hoc* Tukey Honestly Significant Difference (HSD) test was performed, and differences with P < 0.05 (*), P < 0.01 (**), and P < 0.001 (***) were highlighted and considered statistically significant.

### N. Ethics

Ethics approval is not required.

## SUPPLEMENTARY MATERIAL

See the supplementary material File 1 for extended protocols, supplementary material Tables S1, S2, S3, and S4, and supplementary material Figs. S1, S2, S3, S4, and S5. See the supplementary material File 2 for the GO_biological processes of the GSEA analysis. See the supplementary material File 3 for the ISMARA targets of the top 10 TFs. See the supplementary material File 4 for the protein list. See the supplementary material File 5 for the metabolomic analysis.

## Data Availability

The data that support the findings of this study are available within the supplementary material and the corresponding author upon reasonable request.
